# Supplementary material for: Modeling glioblastoma heterogeneity as a dynamic network of cell states
Source: Mol Syst Biol. 2021 Sep 16;17(9):e10105. doi: 10.15252/msb.202010105 (PMC8444284; doi:10.15252/msb.202010105)
Supplement: Supplementary file 5 — Source Data for Figure 3 [file MSB-17-e10105-s001.zip › Figure3A_sourcedata/GSEA_3065/nosigdb_state6.GseaPreranked.1622718443993/NB_KI_CLUSTER1.html]

Details for gene set NB\_KI\_CLUSTER1[GSEA]

|  || Dataset | state6 |
| Phenotype | NoPhenotypeAvailable |
| Upregulated in class | na\_pos |
| GeneSet | NB\_KI\_CLUSTER1 |
| Enrichment Score (ES) | 0.9429265 |
| Normalized Enrichment Score (NES) | 3.1548874 |
| Nominal p-value | 0.0 |
| FDR q-value | 0.0 |
| FWER p-Value | 0.0 |
Table: GSEA Results Summary

  

Fig 1: Enrichment plot: NB\_KI\_CLUSTER1      
 Profile of the Running ES Score & Positions of GeneSet Members on the Rank Ordered List

  

| PROBE | GENE SYMBOL | GENE\_TITLE | RANK IN GENE LIST | RANK METRIC SCORE | RUNNING ES | CORE ENRICHMENT || 1 | RPLP1 |  |  | 6 | 0.329 | 0.0445 | Yes |
| 2 | EEF1A1 |  |  | 9 | 0.325 | 0.0889 | Yes |
| 3 | RPS27 |  |  | 11 | 0.307 | 0.1309 | Yes |
| 4 | RPS19 |  |  | 14 | 0.295 | 0.1712 | Yes |
| 5 | RPL13 |  |  | 21 | 0.286 | 0.2097 | Yes |
| 6 | RPL3 |  |  | 22 | 0.285 | 0.2488 | Yes |
| 7 | RPLP2 |  |  | 31 | 0.271 | 0.2850 | Yes |
| 8 | RPL10 |  |  | 33 | 0.265 | 0.3213 | Yes |
| 9 | RPL18A |  |  | 39 | 0.252 | 0.3553 | Yes |
| 10 | RPL34 |  |  | 41 | 0.251 | 0.3896 | Yes |
| 11 | RPL41 |  |  | 42 | 0.249 | 0.4238 | Yes |
| 12 | TPT1 |  |  | 48 | 0.247 | 0.4570 | Yes |
| 13 | RPS8 |  |  | 51 | 0.245 | 0.4904 | Yes |
| 14 | RPS4X |  |  | 58 | 0.238 | 0.5224 | Yes |
| 15 | RPS3 |  |  | 59 | 0.238 | 0.5550 | Yes |
| 16 | RPL28 |  |  | 62 | 0.235 | 0.5870 | Yes |
| 17 | RPL32 |  |  | 69 | 0.231 | 0.6180 | Yes |
| 18 | RPS3A |  |  | 80 | 0.224 | 0.6476 | Yes |
| 19 | RPL39 |  |  | 82 | 0.223 | 0.6781 | Yes |
| 20 | RPS25 |  |  | 84 | 0.223 | 0.7086 | Yes |
| 21 | RPS7 |  |  | 107 | 0.210 | 0.7349 | Yes |
| 22 | RPS27A |  |  | 136 | 0.198 | 0.7588 | Yes |
| 23 | RPS2 |  |  | 156 | 0.189 | 0.7826 | Yes |
| 24 | RPS12 |  |  | 164 | 0.187 | 0.8074 | Yes |
| 25 | RPS15A |  |  | 165 | 0.187 | 0.8330 | Yes |
| 26 | RPS29 |  |  | 170 | 0.185 | 0.8579 | Yes |
| 27 | RPL27A |  |  | 183 | 0.182 | 0.8815 | Yes |
| 28 | RPS18 |  |  | 185 | 0.181 | 0.9062 | Yes |
| 29 | RPL23A |  |  | 246 | 0.163 | 0.9217 | Yes |
| 30 | RPL13A |  |  | 255 | 0.161 | 0.9429 | Yes |
| 31 | RPL21 |  |  | 554 | 0.118 | 0.9251 | No |
| 32 | RPS6 |  |  | 647 | 0.110 | 0.9297 | No |
Table: GSEA details [plain text format]

  

Fig 2: NB\_KI\_CLUSTER1: Random ES distribution      
 Gene set null distribution of ES for **NB\_KI\_CLUSTER1**
